# Supplementary material for: A fault-intrusion-tolerant system and deadline-aware algorithm for scheduling scientific workflow in the cloud
Source: PeerJ Comput Sci. 2021 Nov 2;7:e747. doi: 10.7717/peerj-cs.747 (PMC8576554; doi:10.7717/peerj-cs.747)
Supplement: Supplemental Information 6 [file peerj-cs-07-747-s006.zip › Myproject1.1/docs/org/cloudbus/cloudsim/ResCloudlet.html]

ResCloudlet


JavaScript is disabled on your browser.


- Overview
- Package
- Class
- Use
- Tree
- Deprecated
- Index
- Help

- Prev Class
- Next Class

- Frames
- No Frames

- All Classes

- Summary:
- Nested |
- Field |
- Constr |
- Method

- Detail:
- Field |
- Constr |
- Method


org.cloudbus.cloudsim

## Class ResCloudlet

- java.lang.Object
- - org.cloudbus.cloudsim.ResCloudlet

- ---

    

  ```
  public class ResCloudlet
  extends java.lang.Object
  ```

  CloudSim ResCloudlet represents a Cloudlet submitted to CloudResource for processing. This class
  keeps track the time for all activities in the CloudResource for a specific Cloudlet. Before a
  Cloudlet exits the CloudResource, it is RECOMMENDED to call this method
  `finalizeCloudlet()`.

  It contains a Cloudlet object along with its arrival time and the ID of the machine and the Pe
  (Processing Element) allocated to it. It acts as a placeholder for maintaining the amount of
  resource share allocated at various times for simulating any scheduling using internal events.

  Since:
  :   CloudSim Toolkit 1.0

- - ### Constructor Summary

    Constructors

    Constructor and Description

    `ResCloudlet(Cloudlet cloudlet)`

    Allocates a new ResCloudlet object upon the arrival of a Cloudlet object.

    `ResCloudlet(Cloudlet cloudlet,
    long startTime,
    int duration,
    int reservID)`

    Allocates a new ResCloudlet object upon the arrival of a Cloudlet object.
  - ### Method Summary

    Methods

    | Modifier and Type | Method and Description |
    | --- | --- |
    | `void` | `finalizeCloudlet()` Finalizes all relevant information before exiting the CloudResource entity. |
    | `double` | `getClouddletFinishTime()` Gets the Cloudlet's finish time. |
    | `Cloudlet` | `getCloudlet()` Gets this Cloudlet object. |
    | `double` | `getCloudletArrivalTime()` Gets arrival time of a cloudlet. |
    | `int` | `getCloudletClassType()` Gets the Cloudlet's class type. |
    | `int` | `getCloudletId()` Gets this Cloudlet entity Id. |
    | `long` | `getCloudletLength()` Gets the Cloudlet's length. |
    | `int` | `getCloudletStatus()` Gets the Cloudlet status. |
    | `long` | `getCloudletTotalLength()` Gets the total Cloudlet's length (across all PEs). |
    | `int` | `getDurationTime()` Gets the reservation duration time. |
    | `double` | `getExecStartTime()` Gets the Cloudlet's execution start time. |
    | `int` | `getMachineId()` Gets machine ID. |
    | `int[]` | `getMachineIdList()` Gets a list of Machine IDs. |
    | `int` | `getNumberOfPes()` Gets the number of PEs required to execute this Cloudlet. |
    | `int` | `getPeId()` Gets Pe ID. |
    | `int[]` | `getPeIdList()` Gets a list of Pe IDs. |
    | `long` | `getRemainingCloudletLength()` Gets the remaining cloudlet length. |
    | `int` | `getReservationID()` Gets the reservation ID that owns this Cloudlet. |
    | `long` | `getStartTime()` Gets the Cloudlet or reservation start time. |
    | `java.lang.String` | `getUid()` Get unique string identificator of the VM. |
    | `int` | `getUserId()` Gets the user or owner of this Cloudlet. |
    | `boolean` | `hasReserved()` Checks whether this Cloudlet is submitted by reserving or not. |
    | `boolean` | `setCloudletStatus(int status)` Sets the Cloudlet status. |
    | `void` | `setExecParam(double wallClockTime, double actualCPUTime)` Sets this Cloudlet's execution parameters. |
    | `void` | `setFinishTime(double time)` Sets the finish time for this Cloudlet. |
    | `void` | `setMachineAndPeId(int machineId, int peId)` Sets the machine and Pe (Processing Element) ID. |
    | `void` | `updateCloudletFinishedSoFar(long miLength)` A method that updates the length of cloudlet that has been completed. |

    - ### Methods inherited from class java.lang.Object

      `clone, equals, finalize, getClass, hashCode, notify, notifyAll, toString, wait, wait, wait`

- - ### Constructor Detail


    - #### ResCloudlet

      ```
      public ResCloudlet(Cloudlet cloudlet)
      ```

      Allocates a new ResCloudlet object upon the arrival of a Cloudlet object. The arriving time
      is determined by `gridsim.CloudSim#clock()`.

      Parameters:
      :   `cloudlet` - a cloudlet object

      See Also:
      :   `gridsim.CloudSim#clock()`


    - #### ResCloudlet

      ```
      public ResCloudlet(Cloudlet cloudlet,
                 long startTime,
                 int duration,
                 int reservID)
      ```

      Allocates a new ResCloudlet object upon the arrival of a Cloudlet object. Use this
      constructor to store reserved Cloudlets, i.e. Cloudlets that done reservation before. The
      arriving time is determined by `gridsim.CloudSim#clock()`.

      Parameters:
      :   `cloudlet` - a cloudlet object
      :   `startTime` - a reservation start time. Can also be interpreted as starting time to
          execute this Cloudlet.
      :   `duration` - a reservation duration time. Can also be interpreted as how long to execute
          this Cloudlet.
      :   `reservID` - a reservation ID that owns this Cloudlet

      See Also:
      :   `gridsim.CloudSim#clock()`
  - ### Method Detail


    - #### getStartTime

      ```
      public long getStartTime()
      ```

      Gets the Cloudlet or reservation start time.

      Returns:
      :   Cloudlet's starting time


    - #### getDurationTime

      ```
      public int getDurationTime()
      ```

      Gets the reservation duration time.

      Returns:
      :   reservation duration time


    - #### getNumberOfPes

      ```
      public int getNumberOfPes()
      ```

      Gets the number of PEs required to execute this Cloudlet.

      Returns:
      :   number of Pe


    - #### getReservationID

      ```
      public int getReservationID()
      ```

      Gets the reservation ID that owns this Cloudlet.

      Returns:
      :   a reservation ID


    - #### hasReserved

      ```
      public boolean hasReserved()
      ```

      Checks whether this Cloudlet is submitted by reserving or not.

      Returns:
      :   true if this Cloudlet has reserved before, false otherwise


    - #### getCloudletId

      ```
      public int getCloudletId()
      ```

      Gets this Cloudlet entity Id.

      Returns:
      :   the Cloudlet entity Id


    - #### getUserId

      ```
      public int getUserId()
      ```

      Gets the user or owner of this Cloudlet.

      Returns:
      :   the Cloudlet's user Id


    - #### getCloudletLength

      ```
      public long getCloudletLength()
      ```

      Gets the Cloudlet's length.

      Returns:
      :   Cloudlet's length


    - #### getCloudletTotalLength

      ```
      public long getCloudletTotalLength()
      ```

      Gets the total Cloudlet's length (across all PEs).

      Returns:
      :   total Cloudlet's length


    - #### getCloudletClassType

      ```
      public int getCloudletClassType()
      ```

      Gets the Cloudlet's class type.

      Returns:
      :   class type of the Cloudlet


    - #### setCloudletStatus

      ```
      public boolean setCloudletStatus(int status)
      ```

      Sets the Cloudlet status.

      Parameters:
      :   `status` - the Cloudlet status

      Returns:
      :   true if the new status has been set, false otherwise


    - #### getExecStartTime

      ```
      public double getExecStartTime()
      ```

      Gets the Cloudlet's execution start time.

      Returns:
      :   Cloudlet's execution start time


    - #### setExecParam

      ```
      public void setExecParam(double wallClockTime,
                      double actualCPUTime)
      ```

      Sets this Cloudlet's execution parameters. These parameters are set by the CloudResource
      before departure or sending back to the original Cloudlet's owner.

      Parameters:
      :   `wallClockTime` - the time of this Cloudlet resides in a CloudResource (from arrival time
          until departure time).
      :   `actualCPUTime` - the total execution time of this Cloudlet in a CloudResource.


    - #### setMachineAndPeId

      ```
      public void setMachineAndPeId(int machineId,
                           int peId)
      ```

      Sets the machine and Pe (Processing Element) ID.

      Parameters:
      :   `machineId` - machine ID
      :   `peId` - Pe ID


    - #### getMachineId

      ```
      public int getMachineId()
      ```

      Gets machine ID.

      Returns:
      :   machine ID or -1 if it is not specified before


    - #### getPeId

      ```
      public int getPeId()
      ```

      Gets Pe ID.

      Returns:
      :   Pe ID or -1 if it is not specified before


    - #### getPeIdList

      ```
      public int[] getPeIdList()
      ```

      Gets a list of Pe IDs.   
      NOTE: To get the machine IDs corresponding to these Pe IDs, use `getMachineIdList()`.

      Returns:
      :   an array containing Pe IDs.


    - #### getMachineIdList

      ```
      public int[] getMachineIdList()
      ```

      Gets a list of Machine IDs.   
      NOTE: To get the Pe IDs corresponding to these machine IDs, use `getPeIdList()`.

      Returns:
      :   an array containing Machine IDs.


    - #### getRemainingCloudletLength

      ```
      public long getRemainingCloudletLength()
      ```

      Gets the remaining cloudlet length.

      Returns:
      :   cloudlet length


    - #### finalizeCloudlet

      ```
      public void finalizeCloudlet()
      ```

      Finalizes all relevant information before exiting the CloudResource entity. This
      method sets the final data of:
      - wall clock time, i.e. the time of this Cloudlet resides in a CloudResource (from arrival
        time until departure time).- actual CPU time, i.e. the total execution time of this Cloudlet in a CloudResource.- Cloudlet's finished so far


    - #### updateCloudletFinishedSoFar

      ```
      public void updateCloudletFinishedSoFar(long miLength)
      ```

      A method that updates the length of cloudlet that has been completed.

      Parameters:
      :   `miLength` - cloudlet length in Instructions (I)


    - #### getCloudletArrivalTime

      ```
      public double getCloudletArrivalTime()
      ```

      Gets arrival time of a cloudlet.

      Returns:
      :   arrival time


    - #### setFinishTime

      ```
      public void setFinishTime(double time)
      ```

      Sets the finish time for this Cloudlet. If time is negative, then it is being ignored.

      Parameters:
      :   `time` - finish time


    - #### getClouddletFinishTime

      ```
      public double getClouddletFinishTime()
      ```

      Gets the Cloudlet's finish time.

      Returns:
      :   finish time of a cloudlet or -1.0 if it cannot finish in this hourly slot


    - #### getCloudlet

      ```
      public Cloudlet getCloudlet()
      ```

      Gets this Cloudlet object.

      Returns:
      :   cloudlet object


    - #### getCloudletStatus

      ```
      public int getCloudletStatus()
      ```

      Gets the Cloudlet status.

      Returns:
      :   Cloudlet status


    - #### getUid

      ```
      public java.lang.String getUid()
      ```

      Get unique string identificator of the VM.

      Returns:
      :   string uid


- Overview
- Package
- Class
- Use
- Tree
- Deprecated
- Index
- Help

- Prev Class
- Next Class

- Frames
- No Frames

- All Classes

- Summary:
- Nested |
- Field |
- Constr |
- Method

- Detail:
- Field |
- Constr |
- Method
